# Early Exposure to Intermediate-Frequency Magnetic Fields Alters Brain Biomarkers without Histopathological Changes in Adult Mice

**DOI:** 10.3390/ijerph120404406

**Published:** 2015-04-22

**Authors:** Tin-Tin Win-Shwe, Shin Ohtani, Akira Ushiyama, Naoki Kunugita

**Affiliations:** 1 Center for Environmental Health Sciences, National Institute for Environmental Studies, 16-2 Onogawa, Tsukuba, Ibaraki 305-8506, Japan; 2 Department of Environmental Health, National Institute of Public Health, 2-3-6 Minami, Wako City, Saitama 351-0197, Japan; E-Mails: ohtani@niph.go.jp (S.O.); ushiyama@niph.go.jp (A.U.); kunugita@niph.go.jp (N.K.)

**Keywords:** intermediate frequency electromagnetic field, murine developmental period, hippocampus, NMDA receptor, inflammatory markers

## Abstract

Recently we have reported that intermediate-frequency magnetic field (IF-MF) exposure transiently altered the mRNA expression levels of memory function-related genes in the hippocampi of adult male mice. However, the effects of IF-MF exposure during brain development on neurological biomarkers have not yet been clarified. In the present study, we investigated the effect of IF-MF exposure during development on neurological and immunological markers in the mouse hippocampus in 3- and 7-week-old male mice. Pregnant C57BL/6J mice were exposed to IF-MF (21 kHz, 3.8 mT) for one hour per day from organogenesis period day 7 to 17. At adolescence, some IF-MF-exposed mice were further divided into exposure, recovery, and sham-exposure groups. The adolescent-exposure groups were exposed again to IF-MF from postnatal day 27 to 48. The expression of mRNA in the hippocampi was examined using a real-time RT-PCR method, and microglia activation was examined by immunohistochemical analysis. The expression levels of NR1 and NR2B as well as transcription factors (CaMKIV, CREB1), inflammatory mediators (COX2, IL-1 β,TNF-α), and the oxidative stress marker heme-oxygenase (HO)-1 were significantly increased in the IF-MF-exposed mice, compared with the control group, in the 7-week-old mice, but not in the 3-week-old mice. Microglia activation was not different between the control and other groups. This study provides the first evidence that early exposure to IF-MF reversibly affects the NMDA receptor, its related signaling pathways, and inflammatory mediators in the hippocampus of young adult mice; these changes are transient and recover after termination of exposure without histopathological changes.

## 1. Introduction

Major sources of IF-MF include induction heating (IH) cookers, inductively coupled power transmission for industrial material handling machines or home appliances, and a variety of wireless communication systems [1], magnetic resonance imaging machines, induction heaters, and welding machines [2,3]. Currently, cooking appliances based on the principle of electromagnetic induction are being used domestically worldwide. An IH cooking hob mainly generates intermediate-frequency magnetic fields (IF-MF; from 20 kHz to 90 kHz) to heat cooking pans. However, whether electromagnetic fields originating from household appliances represent a health risk remains uncertain. Because of the lack of sufficient data of the biological effects of IF-MF, the World Health Organization (WHO) has recommended the research of IF-MF on biological systems [4]. In addition, studying the biological effects of IF-MF exposure, especially in susceptible individuals such as children, during development is essential for preserving the life of the next generation.

Some of experimental evidence has indicated that there is a positive correlation between magnetic field exposure and an increased risk of cancer and neurological disorders [5,6,7,8]. Recent studies have shown that exposure to extremely low frequency magnetic fields causes behavioral and cognitive disturbances, attention deficit, and impaired spatial learning in rats [9] and induced significant impairments in detour learning and one-trial passive avoidance learning in chicks [10,11]. Some *in vivo* studies have indicated that there is no association between extremely-low-frequency MF and teratogenicity in chick and rat embryo models [12,13]. Recently, we have reported that exposure to 21-kHz sinusoidal IF-MF at 3.8 mT for 1 h/day for 14 days did not affect hematological parameters and immune functions in juvenile rats [14]. Regarding *in vitro* studies, extremely-low-frequency electromagnetic field exposure has been shown to modify the biophysical properties of cell membranes [15] and to affect ion channels, such as calcium channels [16,17] and sodium channels [18]. One *in vitro* study has reported that the IF-MF generated by IH cooking appliances was not associated with genotoxicity [19]. Taken together, exposure to extremely-low-frequency MF showed some behavioral disturbances in experimental animals, however, exposure to IF-MF does not show any apparent biological effects. Determination of the potential neurotoxic effects of IF-MF and an in-depth understanding of its mechanism of action are essential for the safe use of IF-MF-generating household appliances in homes and industries.

Previously, our research group reported that IF-MF exposure transiently affected the expression levels of memory function-related genes in the hippocampi of adult C57BL/6J male mice [20]. However, the effects of IF-MF exposure during development on neurological biomarkers have not yet been clarified. Here, we investigated the effects of IF-MF exposure during the organogenesis and/or adolescent period on the expression levels of memory function-related genes, their related transduction molecules, and inflammatory markers in the hippocampi of 3-week-old and 7-week-old mice.

Developmental stages can be divided into fetal, newborn, infancy, toddler, puberty, adolescence and adulthood. During these developmental stages, the fetal and adolescence periods are potential periods of exposure to IF-MF because of the common usage of a IH cooking hob by pregnant mothers and teenagers preparing meals for their family. Therefore, we examined the effects of IF-MF exposure on neurological and immunological markers in the mouse brain during these developmental stages.

## 2. Materials and Methods

### 2.1. Animals

**Figure 1:**
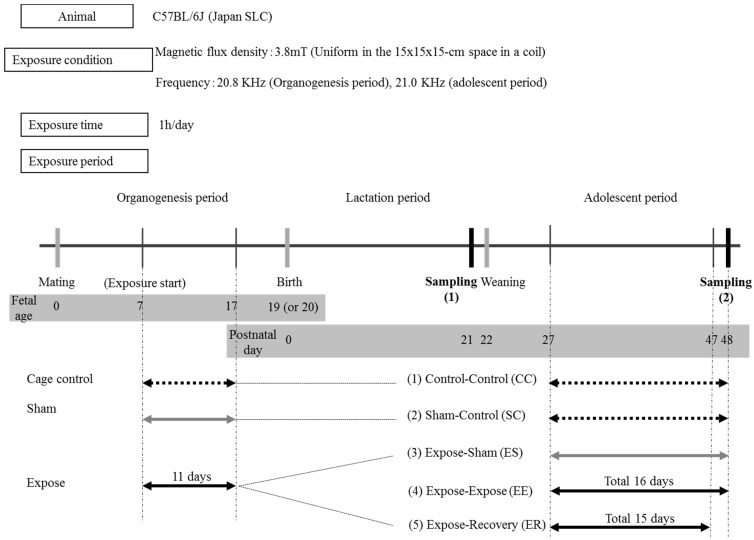
Experimental protocol. Organogenesis and adolescent IF-MF exposure were performed from GD 7 to 17 (total 11 days) and from PND 27 to 48 (Actual experiment period was 22 days which contained 16 exposure days plus 6 week-ends unexposed days), respectively. IF-MF exposure was performed at 21 kHz, 3.8 mT for 1 h per day.

Gestational day (GD) 3 pregnant C57BL/6J mice (vaginal plug was checked) were purchased from Japan SLC Inc. (Shizuoka, Japan). The pregnant mice were allotted to a control, sham, or exposure group and IF-MF was applied at 21 kHz, 3.8 mT, for 1 h per day. At the age of postnatal day (PND) 21, hippocampal samples were obtained from male pups to evaluate the effects of IF-MF exposure during gestation. In some groups, male pups were exposed to IF-MF again from PND 27 to 48 to evaluate the combined effects of organogenesis and adolescent exposure (Figure 1). Mice were fed a commercial diet (FR-2; Funabashi Farm Co., Chiba, Japan) and were given tap water *ad libitum*; the animals were housed in individual cages under controlled environmental conditions (temperature, 23 ± 1 °C; humidity, 50% ± 5%; lights on from 08:00 to 20:00 h). To detect the persistency of the IF-MF effect, we also examined a “recovery group,” which was exposed to IF-MF for 15 days and then left for one day in their home cages before hippocampal sample collection. This study was approved by the Ethics Committee for Experimental Animals of the National Institute of Public Health, Japan.

### 2.2. IF-MF Generation

We used an *in vivo* exposure apparatus that was developed collaboratively with National Institute for Public Health, Japan and Tokyo Metropolitan University, Japan [21,22,23]. The exposure apparatus generates homogeneous IF-MF (±5%) under a sinusoidal condition of 21 kHz and a magnetic flux density of 3.8 mT, with a cubic space of 150 mm on one side. During a 1-h period of exposure to an IF-MF at 3.8 mT, the inside and the surface temperature of the coil were measured using an infrared camera. The temperature inside the exposure device was regulated using a water-cooling circuit system, and the temperature was maintained at 24 ± 1 °C throughout the exposure period.

### 2.3. Exposure to IF-MF

Pregnant mice were placed in the exposure device and were exposed to IF-MF (21 kHz) at 3.8 mT for 1 h per day from gestational day (GD) 7 to 17 (total of 11 days). For the organogenesis exposure, the pregnant mice were allotted to three groups: Control, sham, and exposure. For the adolescent exposure, the pups were allotted to five groups as follows: (1) control-control (CC; control during both developmental periods), (2) sham-control (SC; sham exposure during organogenesis period and control during adolescent period), (3) expose-sham (ES; IF-MF exposure during organogenesis period and sham exposure during adolescent period), (4) expose-recovery (ER; IF-MF exposure during both developmental periods and one day recovery from exposure) and (5) expose-expose (EE; IF-MF exposure during organogenesis period and exposure during adolescent period (PND 27 to 48 except weekends, total of 16 days)).

We used six mice each for exposure, sham or control in in utero exposed group and six mice each for CC, SC, ES, EE or ER groups in 7 week-old mice. IF-MF exposure was performed as described previously [20]. Briefly, the mice were placed in a rounded acryl cage containing six divisions for the simultaneous exposure of six mice without restraint, and the cage was placed inside the coil during exposure. The IF-MF generated by the coils was classified as a vertical field, since the field lines were perpendicular to the bottom plane of the cage. Therefore, the IF-MF was applied in a dorsal-ventral direction relative to each the mouse’s body.

### 2.4. Quantification of mRNA Expression Levels

At an age of 3 weeks or at 1 h (for exposure group) or 24 h (for recovery group) after the completion of the final IF-MF exposure during the adolescent period (7-week-old mice), the mice were sacrificed under deep pentobarbital anesthesia and the hippocampi were collected from each group of mice and frozen quickly in liquid nitrogen, then stored at –80 °C until the extraction of the total RNA as described previously [24]. Briefly, the total RNA was extracted from the hippocampal samples using the BioRobot EZ-1 and EZ-1 RNA tissue mini kits (Qiagen GmbH, Hilden, Germany). Then, the purity of the total RNA was examined, and the quantity was estimated using the ND-1000 NanoDrop RNA Assay protocol (NanoDrop, Wilmington, DE, USA), as described previously [21]. Next, we performed first-strand cDNA synthesis from the total RNA using SuperScript RNase H^−^Reverse Transcriptase II (Invitrogen, Carlsbad, CA, USA), according to the Manufacturer’s protocol. We examined the hippocampal mRNA expression levels using a quantitative real-time RT-PCR method and the Applied Biosystems (ABI) Prism 7000 Sequence Detection System (Applied Biosystems Inc., Foster City, CA, USA). The tissue 18S rRNA level was used as an internal control. The primer sequences used in the present study are shown below. Some primers (cFos, NM_010234; NR1, NM_008169; NR2A, NM_008170; NR2B, NM_008171; BDNF, NM_007540; IL-1 β, NM_008361; COX2, NM_011198; HO1, NM_010442) were purchased from Qiagen, Sample and Assay Technologies. Other primers were designed in our laboratory as follows: 18S (forward 5'-TACCACATCCAAAAGGCAG-3', reverse 5'-TGCCCTCCAA TGGATCCTC-3'), CaMKIV (forward 5'-AAATCAGCCTGGTCCTTGAG-3', reverse 5'-TCTGGTTTGA GGTCACGATG-3'), CREB1 (forward 5'-GGAATCTGGAGCAGACAACC-3', reverse 5'-ATAACGCCAT GGACCTGGAC-3'), NGF (forward 5'-TGGGCTTCAGGGACAGAGTC-3', reverse 5'-CAGCTTTCTAT ACTGGCCGCAG-3'), and TNF-α (forward 5’-GGTTCCTTTGTGGCACTTG-3', reverse 5'-TTCTCTTG GTGACCGGGAG-3'). Data were analyzed using the comparative threshold cycle method. Then, the relative mRNA expression levels were expressed as mRNA signals per unit of 18S rRNA expression.

### 2.5. Histological Examination

We performed the histological examination as described previously [20]. The brains were removed from two mice from each of the control and exposure groups after the animals had been deeply anesthetized with sodium pentobarbital; the brains were then fixed with 10% formalin. The fixed brains were dehydrated using a graded series of ethanol, cleared with xylene, and embedded in paraffin. Coronal paraffin sections were cut at a thickness of 7 μm using a microtome and were mounted on 3-aminopropyltriethoxysilane-coated glass slides. Each section was stained with hematoxylin and eosin (H&E) for histological examination.

### 2.6. Immunohistochemistry

Microglia activation indicates a neurotoxic effect in the brain. To detect microglia activation in the hippocampus, the slides were immunostained with microglia marker Iba1. Briefly, the brain sections were immersed in absolute ethanol followed by 10% H_2_O_2_ for 10 min each at room temperature. After rinsing in 0.01-M phosphate buffer saline, the sections were blocked with 2% normal swine serum in PBS for 30 min at room temperature and then reacted with goat polyclonal anti-Iba1 (diluted 1:100; abcam: ab5076; Tokyo, Japan) in PBS for 1 h at 37 °C. The sections were reacted with biotinylated donkey anti-rabbit IgG (1:300 Histofine; Nichirei Bioscience, Tokyo, Japan) in PBS for 1 h at 37 °C before and after rinsing in PBS. The sections were then incubated with peroxidase-tagged streptavidin (1:300, ABC KIT) containing PBS for 1 h at room temperature. After rinsing in PBS, Iba1 immunoreactivity was detected using a Dako DAB Plus Liquid System (Dako Corp. Carpinteria, CA, USA). To determine the immunoreactivity of Iba1 in the hippocampus, photomicrographic digital images (150 dpi, 256 scales) of the hippocampal regions were taken using a CCD camera connected to a light microscope.

### 2.7. Statistical Analysis

All the data were expressed as the mean ± standard error (S.E.). The statistical analysis was performed using the StatMate II statistical analysis system for Microsoft Excel, Version 5.0 (Nankodo Inc., Tokyo, Japan). The data were analyzed using a one-way analysis of variance with a post-hoc analysis using the Bonferroni/Dunn method. Differences were considered significant at *p* < 0.05.

## 3. Results

### 3.1. Body Weight and Organ Weights

To determine the general toxicity of IF-MF exposure, we measured the body and organ weights of the male mice after the completion of the exposure periods at the time of sampling (3 weeks or 7 weeks of age). Among the organogenesis-exposure groups, the body weight was significantly higher in the exposure group than in the control group; the spleen weight of the mice in the exposure group increased in parallel with the body weight and the thymus weight did not differ among the groups in in utero exposed male mice. Among the adolescent-exposure groups, no significant differences in the body weight or the relative weights of the spleen and thymus were observed among the groups in the 7-week old male mice (data not shown).

### 3.2. Immediate Early Gene, Memory Function-Related Genes, Their Signal Transduction Pathway Genes and Neurotrophins in the Hippocampus

**Figure 2:**
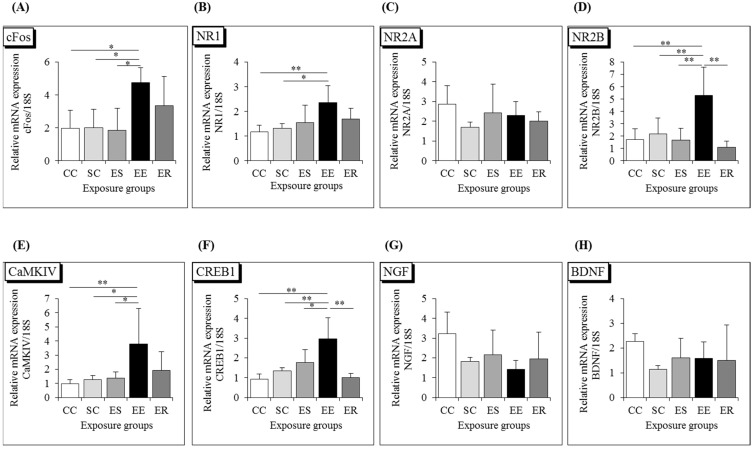
Messenger RNA expression level of (**A**) immediate early gene c-Fos, NMDA receptor subunits: (**B**) NR1, (**C**) NR2A, (**D**) NR2B, transcription factor: (**E**) CaMKIV, (**F**) CREB1, neurotrophins: (**G**) NGF, (**H**) BDNF in the hippocampi of CC, SC, EE, ES and ER groups of 7-week-old male mice. Each bar represents the mean ± SD (*n* = 6, ** *p* < 0.01, * *p* < 0.05). CC, control-control; SC, sham-control; EE, exposed-exposed; ES, exposed-sham; ER, exposed-recovery in organogenesis and adolescent periods respectively.

The expression of the immediate early gene c-Fos is an indirect marker of neuronal activity because c-fos is often expressed when neurons are activated [25,26]. The upregulation of c-Fos mRNA in a neuron indicates recent activity [27]. First, we detected the expressions of early genes to determine whether any changes occurred in the hippocampus after IF-MF exposure. The early gene c-Fos expression was significantly increased in the EE group, compared with the other groups (CC, SC, ES) (Figure 2A, *p* < 0.05).

Our previous study indicated that IF-MF exposure transiently affected the expression levels of memory function-related genes in the hippocampus of adult C57BL/6J male mice. In the present study, we investigated the effects of developmental IF-MF exposure on memory function-related genes such as N-methyl-d-aspartate (NMDA) subunits, their transduction genes, neurotrophic factors, and proinflammatory cytokines in the hippocampi of young adult mice.

The expression of NR1 mRNA was significantly higher in the EE group than in the CC and SC groups, the expression of NR2B mRNA was significantly higher in the EE group than in the other groups, while the expression of the NR2A mRNA did not differ among the five treated groups. (Figure 2B,D, ** *p* < 0.01; * *p* < 0.05).

Next, we examined the signal transduction pathway of NMDA receptors, such as CaMKIV and CREB1, in the hippocampus and found that the expressions of CaMKIV and CREB1 were elevated in the IF-MF-exposed (EE) group, in parallel with the expressions of the NMDA subunits NR1 and NR2B (Figure 2E,F, ** *p* < 0.01; * *p* < 0.05). However, the expressions of neurotrophins, such as NGF and BDNF, did not differ between the control and other groups (Figure 2G,H).

### 3.3. Proinflammatory Cytokines and Oxidative Stress Markers in the Hippocampus

**Figure 3:**
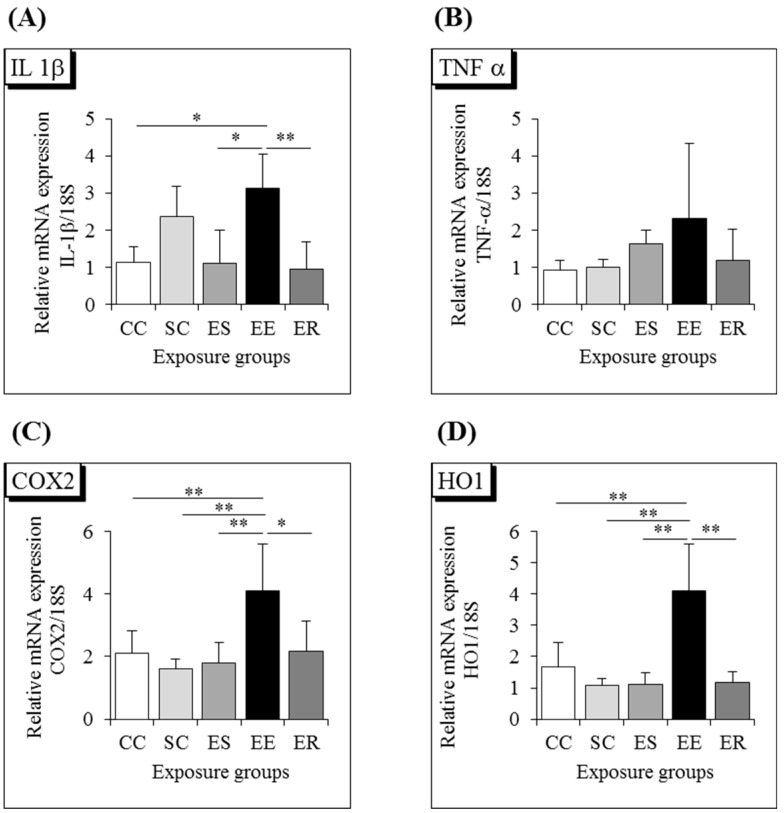
Messenger RNA expression level of proinflammatory cytokines (**A**) IL-1β, (**B**) TNF-α, the inflammatory marker (**C**) COX2, and the oxidative stress marker (**D**) HO1 in the hippocampi of CC, SC, EE, ES, and ER groups of 7-week-old male mice. Each bar represents the mean ± SD (*n* = 6, ** *p* < 0.01, * *p* < 0.05).

To detect the inflammatory condition in the brain, we investigated the expression levels of potent inflammatory cytokines, such as IL-1 β and TNF-α. The expression levels of IL-1 β and TNF-α were higher in the IF-MF-exposed (EE) group than in the other groups (Figure 3A,B, ** *p* < 0.01; * *p* < 0.05). Cyclooxygenase (COX) is the enzyme responsible for the conversion of arachidonic acid to prostaglandin, which is involved in the inflammatory response. COX2 is an inducible form and is released at the site of inflammation. In the present study, COX2 mRNA was remarkably upregulated in the IF-MF-exposed (EE) group, compared with the other groups (Figure 3C, ** *p* < 0.01). To understand the mechanism underlying the inflammatory response in the hippocampi of mice exposed to IF-MF, we also examined the expression of the oxidative stress marker HO1 and found that HO1 mRNA was significantly upregulated in the IF-MF-exposed (EE) group, compared with the other groups (Figure 3D, ** *p* < 0.01).

### 3.4. Effect of IF-MF Exposure on Histology of Hippocampus

**Figure 4:**
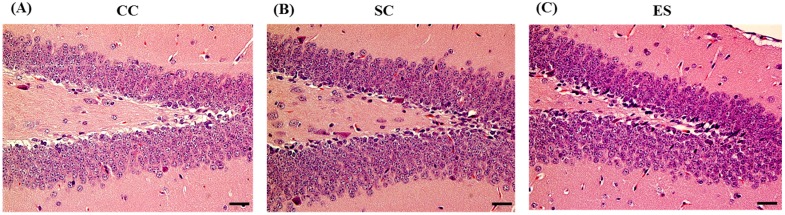

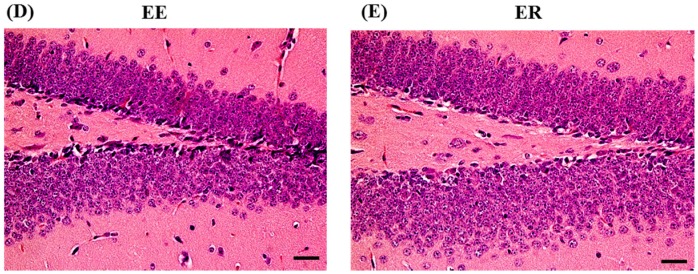
Representative photomicrographs showing the histology of the hippocampal DG region in (**A**) CC, (**B**) SC, (**C**) EE, (**D**) ES, and (**E**) ER groups (H&E staining) in 7-week-old male mice. Scale bar = 50 μm.

We investigated the morphological changes of hippocampi from control and IF-MF-exposed mice using H & E staining. We did not observe any remarkable morphological changes between the control and the IF-MF-exposed mice (Figure 4).

### 3.5. Effect of IF-MF Exposure on Microglia Activation in the Hippocampus

**Figure 5:**
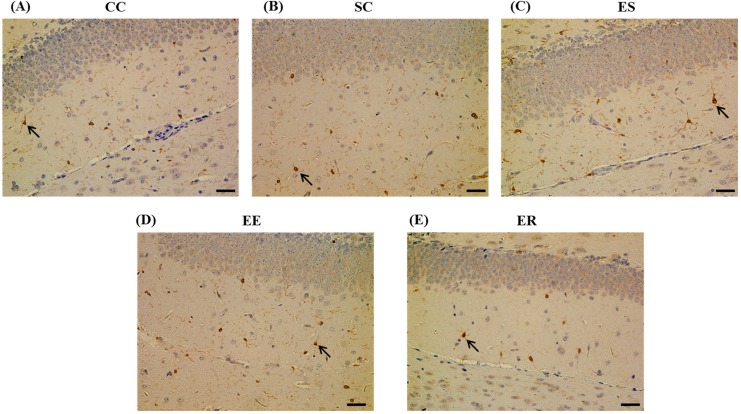
Representative digital photomicrographs of microglia marker Iba1-immunostained sections taken from the hippocampus in (**A**) CC, (**B**) SC, (**C**) EE, (**D**) ES, and (**E**) ER groups of 7-week-old male mice. (Scale bar = 25 μm). Arrow indicates the activated microglia.

Microglia are a major immune cell type in the brain, and activated microglia are involved in neurotoxicity. Using the microglia marker Iba1, we examined microglia activation but did not observe any significant differences between the control and other groups (Figure 5).

The summarized results are presented in Table 1.

**Table 1:** Effect of IF-MF exposure during organogenesis period and/or adolescent period on mRNA expression of neurological and immunological biomarkers on in utero exposed male mice and 7-week-old male mice.

Age	*In utero* Exposure	7-Week-Old	7-Week-Old
Biomarker	Organogenesis Exposure	Organogenesis and Adolescent Exposure (EE)	Organogenesis and Adolescent Exposure (ER)
c-Fos	−	+	−
NR1	+	+	−
NR2A	−	−	−
NR2B	+	+	#
CaMKIV	−	+	−
CREB1	−	+	#
NGF	−	−	−
BDNF	−	−	−
IL-1β	−	+	#
TNF-α	−	+	−
COX2	−	+	#
HO1	−	+	#

− indicates no significant difference compared with the control group; + indicates significantly increased expression compared with the control group; # indicates significantly decreased expression compared with EE group and not different from the control group.

## 4. Discussion

Our present findings provide the first evidence that gestational and adolescence exposure to IF-MF reversibly affects the expression level of NMDA receptor, its related signaling pathways, and inflammatory mediators in the hippocampus of young adult mice. These changes may be transient and may recover after the termination of IF-MF exposure. We found that organogenesis exposure alone did not affect the neurological and immunological biomarkers, except for the expressions of the NMDA receptors NR1 and NR2B, in the mouse hippocampus, and combined exposure during the organogenesis and adolescence periods reversibly affected these biomarkers in young adult mice. We suggest that IF-MF may induce the release of glutamate (an excitatory amino acid neurotransmitter and a ligand for NMDA receptors) from neurons and the release of toxic substances, such as inflammatory mediators, and oxidative stress from microglia, possibly triggering neuroinflammation and/or cognitive impairment.

The brain continues to grow and develop into young adulthood. It was shown that exposure during pre-mating and pre-implantation period has not been proven affecting reproductive functions in rodents [28,29]. In the present study, we have focused on the developmental period exposure on brain cognitive function related biomarkers and we considered to examine the second and third period of gestation during which neural development for cognitive functions such as myelination, glial cell formation, synapse formation, axon and dendritic sprouting are occurred. Moreover, a brain growth spurt occurs in adolescence, and during that period, the brain once again undergoes synapse pruning, similar to the growth that occurs in the brains of infants and toddlers. Increased myelination and rapid neural processing also occur during the transitional period from the teenage years to young adulthood. Developmental periods are thought to represent a time of increased susceptibility to environmental stimuli, such as chemical, radiation and magnetic fields. Exposure to IF-MF during different developmental stages may trigger different biological effects. In the present study, we focused on the fetal and adolescence periods because of the common usage of IH cooking hobs by pregnant mothers and teenagers preparing meals for their family.

Actually, we targeted to assess the neurotoxic markers and inflammatory markers in the brain after exposure to IF-MF. Glutamate is a ligand for NMDA receptor and an excitatory amino acid neurotransmitter and one of the neurotoxic markers in the brain. Real time glutamate level can be assessed by *in vivo* microdialysis method. In the present study, according to the condition of the exposure system, we could not perform *in vivo* microdialysis method. Thus, we considered to examine the one of glutamate receptor, NMDA and then assessed NMDA receptor subunit mRNA levels in the mice brain. Another reason to examine the mRNA level is our previous data are showing mRNA levels in the adult mice brain after IF-MF exposure [20]. Regarding the relationship between NMDA receptors and human health risk: We aimed to investigate the neurotoxicity especially cognitive function in mice after IF-MF exposure, NMDA receptors and signal transduction genes may reflect the cognitive functions in human. Although it is difficult to apply exactly the data from rodents to human, we need to do animal experiment because we could not manipulate human as an experimental model. Thus, we considered to examine the mRNA expression level of NMDA receptor to trigger the potential cognitive deficit in human. We have a plan to analyze protein synthesis/inhibitor enzyme activity, alterations in the functional protein levels, associated cell activation/migration/proliferation, and perform behavioral tests for cognitive functions in our future studies.

The hippocampus is essential for learning and memory functions, and synaptic transmission in the hippocampus is mediated by glutamate receptors. NMDA is a type of glutamate receptor and is important for synaptic plasticity and learning as well as memory functions [30,31]. The excitability of hippocampal neurons are affected by MF exposure, possibly because of the involvement of intracellular calcium channels [32]. Therefore, in the present study, we focused on the expression of NMDA and its signal transduction pathway genes in the hippocampus to detect the effects of IF-MF exposure.

Reportedly, perinatal exposure to rotating magnetic fields (1.5 or 3.0 mT) for 3 days before birth until 3 days after birth significantly reduced the neuronal density in the medial preoptic nucleus of male rats [33]. In addition, the prenatal exposure of pregnant dams to extremely low 7-Hz magnetic fields from 2 days before birth until 14 days after birth can cause behavioral deficits in their offspring through the modulation of nitric oxide (NO), and these deficits can persist into adulthood [34]. However, Nishimura and colleagues reported that no association exists between 20-kHz MF exposure and teratogenicity in chick and rat embryo models [12,13]. Recently, we have also reported that juvenile rats exposed to the 21-kHz IF-MF did not show any effect on the immunological functions and the blood parameters [14]. To the best of our knowledge, very few reports have studied the effects of IF-MF exposure during different developmental periods, especially during the brain growth spurt that occurs during adolescence. This situation prompted us to perform the present study.

In the present study, we examined intermediate-frequency magnetic fields because most household appliances, including IH cooking hobs, generate magnetic fields of 20 kHz to 90 kHz. First, we searched for possible changes in neuronal activity in the hippocampus by evaluating the immediate early genec-Fos. After confirming the presence of changes in neuronal activity in the hippocampus, we examined hippocampus-dependent memory function-related genes, their signal transduction genes, inflammatory markers, and oxidative stress markers. We found that the expression levels of memory function-related genes such as NMDA receptors (NR1, NR2B) and their signal transduction pathway molecules (CaMKIV, CREB1) were significantly upregulated in the hippocampus of 7-week-old male mice exposed to IF-MF during organogenesis and adolescent periods. As mentioned in our previous study of IF-MF exposure in adult mice [16], the upregulation of NMDA receptors and their signal transduction pathway molecules might be caused by excitotoxicity arising from an increase in the neurotransmitter glutamate, which abnormally activates NMDA receptors; another possibility is a compensatory response to a reduction in functional receptors.

Brain inflammation is an important factor in the pathogenesis of neuropsychiatric diseases such as autism spectrum disorders [35,36], which can be triggered by environmental stress such as infection, trauma, or toxic exposure during developmental periods [37,38]. In the present study, the expressions of the inflammatory cytokines IL-1 β and TNF-α were remarkably increased in mice exposed to IF-MF during both their organogenesis and adolescent periods. COX is an enzyme responsible for the formation of prostaglandin, and COX2 is a potent inflammatory mediator that is present at the site of inflammation. We also observed the increased expression of an inflammatory marker (COX2) and an oxidative stress marker (HO1) in parallel with the neurological markers and inflammatory cytokines. Therefore, we speculated that oxidative stress induced by IF-MF exposure might trigger neuroinflammation and NMDA receptor functions in the hippocampus.

One *in vivo* study showed that exposure to 20-kHz MF from GD 0 to 19 in C57BL/6J mice affected neuronal and glial markers in specific brain regions in PND 21 mice [39]. In the present study, although the data were not shown, the mRNA expressions of the NMDA receptor subunits NR1 and NR2B were significantly upregulated in 3-week-old mice. These data suggest that the effects of IF-MF exposure during the organogenesis period may persist until the weaning period. In addition, the adolescent exposure groups showed that IF-MF induced the up-regulation of NMDA receptors (NR2B) and their signal transduction gene (CREB1) as well as inflammatory mediators (IL-1 β, COX2, HO1) in the EE group but returned to the control level in the ER group. These data indicate that one day after the termination of exposure, the expressions of the neuroimmune mediators may have returned to a normal level. Our data suggest that the effects of IF-MF exposure during the organogenesis period persist until the weaning period; in contrast, the animals had recovered from the effects of IF-MF exposure during the adolescent period at one day after the termination of IF-MF exposure. We cannot explain these different effects. One possible explanation is that neuronal cells might function differently during different developmental periods. Alternatively, the neuronal and immune cell turnover rates and resistance to external stress might contribute to the different effects.

Male deer mice are reportedly more sensitive to magnetic stimuli than females [40], and some sexually dimorphic structures may be permanently and differentially affected when exposed perinatally to relatively weak, extremely low-frequency magnetic fields [33]. In addition, sex differences in the behavior of deer mice were observed after exposure to magnetic fields, and a significantly enhanced novel taste preference was observed in male mice, but not in female mice [41]. Taken together, these findings suggest that IF-MF may exert a gender-related influence on brain activity and may be related to the sensitivity of male rodents and the resistance to stress and anxiety of female rodents.

Neurotrophins including NGF and BDNF support the survival of existing neurons, and encourage the growth and differentiation of new neurons and synapses. In addition, neurotrophins and their related receptors have been suggested as targets for neurotoxicants and are known to play a role in bidirectional signaling between neurons and immune cells. Our present study showing that the expression levels of NGF and BDNF were not different between the control and exposed mice. Moreover, we did not observe any significant difference in microglial activation, which is an indicator of neurotoxicity, between the control and IF-MF-exposed mice.

From our findings, we suggest that IF-MF exposure during the organogenesis period alone did not affect neuro-immune biomarkers in the hippocampus of male mice. IF-MF exposure during the adolescent period alone could affect neuro-immune biomarkers in the hippocampus; however, these effects were transient and returned to the control level after the exposure was terminated. The intensity of the IF-MF, the alteration of cell membrane integrity, transport systems and calcium and other ion channel characteristics might be involved in the mechanism of action of IF-MF [42,43,44]. Some recent studies have reported that oxidative stress [45] and nitric oxide may be involved in the effects of EMF exposure [34].

## 5. Conclusions

In conclusion, our findings suggest that developmental exposure to IF-MF may induce neuroinflammation and changes in memory function related genes such as NMDA receptor and related signaling pathway genes by modulating cytokines and excitotoxic glutamate (a ligand for NMDA receptor)-related pathways in IF-MF-exposed mice. We suggest that evaluation of glutamate level in the hippocampus and behavioral assessment will be helpful for evidence of neurotoxic effects after exposure to IF-MF.

Further studies are necessary to clarify the effects of IF-MF exposure during other brain developmental stages. This study provides evidence that early exposure to IF-MF during organogenesis and adolescence periods reversibly affects NMDA receptors, their related signaling pathways, and inflammatory mediators in the hippocampus of young adult mice. Our results also indicate that these changes are transient and recovered after the termination of exposure to IF-MF.
